# Target Repression Induced by Endogenous microRNAs: Large Differences, Small Effects

**DOI:** 10.1371/journal.pone.0104286

**Published:** 2014-08-20

**Authors:** Ana Kozomara, Suzanne Hunt, Maria Ninova, Sam Griffiths-Jones, Matthew Ronshaugen

**Affiliations:** Faculty of Life Sciences, The University of Manchester, Manchester, United Kingdom; University of Oxford, United Kingdom

## Abstract

MicroRNAs are small RNAs that regulate protein levels. It is commonly assumed that the expression level of a microRNA is directly correlated with its repressive activity – that is, highly expressed microRNAs will repress their target mRNAs more. Here we investigate the quantitative relationship between endogenous microRNA expression and repression for 32 mature microRNAs in *Drosophila melanogaster* S2 cells. In general, we find that more abundant microRNAs repress their targets to a greater degree. However, the relationship between expression and repression is nonlinear, such that a 10-fold greater microRNA concentration produces only a 10% increase in target repression. The expression/repression relationship is the same for both dominant guide microRNAs and minor mature products (so-called passenger strands/microRNA* sequences). However, we find examples of microRNAs whose cellular concentrations differ by several orders of magnitude, yet induce similar repression of target mRNAs. Likewise, microRNAs with similar expression can have very different repressive abilities. We show that the association of microRNAs with Argonaute proteins does not explain this variation in repression. The observed relationship is consistent with the limiting step in target repression being the association of the microRNA/RISC complex with the target site. These findings argue that modest changes in cellular microRNA concentration will have minor effects on repression of targets.

## Introduction

MicroRNAs are non-protein-coding RNAs involved in gene regulation at the post-transcriptional level. First discovered as critical regulators of developmental timing in *Caenorhabditis elegans* [1], microRNAs have since been found to function in almost all cellular processes in animals and plants [2], [3]. MicroRNAs are approximately 22 nucleotides in length, generated from large primary transcripts that form imperfect stem loop structures. MicroRNAs regulate gene expression by binding to target mRNAs and promoting transcript degradation or inhibiting translation [4].

MicroRNA biogenesis is orchestrated by a well-conserved set of proteins and enzymes. In animals, the nuclear RNase III enzyme, Drosha, cuts the primary (pri-) microRNA to leave a ∼70 nt hairpin sequence called the precursor (pre-) microRNA [5]–[7]. Exportin-5 then exports the pre-microRNA out of the nucleus where it is cleaved by the cytoplasmic RNase III enzyme Dicer-1 and its partner Loquacious (Loqs) to form a ∼22 nt double-stranded duplex [7]–[9]. In order to repress a target mRNA, the microRNA duplex associates with an Argonaute (Ago) family protein, which constitutes the core of the RNA-induced silencing complex (RISC) [10]. The arm of the duplex that is incorporated into RISC mediates association with target mRNAs by complementary base pairing [4]. MicroRNAs and short interfering RNAs (siRNAs) share much of the same pathway, but differ to some degree in their use of specific Dicer and Argonaute protein paralogs. For example, in *Drosophila melanogaster*, Dicer-1 and Ago-1 are mainly associated with microRNAs, whilst Dicer-2 and Ago-2 are associated with siRNAs. However, some microRNAs have been found to be significantly associated with Ago-2 [11].

Both strands of the mature microRNA duplex have the potential to bind and regulate target transcripts [12]. However, in a given cell type or developmental stage, one strand will often accumulate at significantly higher levels. This is normally assumed to be the functional dominant microRNA [13], [14]. The minor mature product, the so-called microRNA* sequence, is often assumed to be preferentially degraded. There are now several lines of evidence to support the notion that mature microRNA sequences derived from both arms of the hairpin precursor are functional. Firstly, studies in both human cell lines and *Drosophila* embryos have demonstrated that some microRNA* sequences are able to repress target sequences [12], [15], [16]. Secondly, presumed microRNA* strands have been shown to be more highly expressed in various organisms and at various developmental stages [17], [18]. For example, both stands of miR-10 are expressed in *D. melanogaster* and the beetle *Tribolium castaneum*, with the dominant mature microRNA switched between the two species [19], [20].

The cellular concentration of a microRNA is commonly expected to closely relate to its repressive activity, and indeed is often used as a proxy for activity: a highly expressed microRNA will repress the translation of its target mRNAs more and therefore be more functionally important than a less well expressed microRNA. A common corollary is that the strand of the microRNA duplex that is seen to be more highly expressed is assumed to be the functional mature microRNA [21], [22]. The presumption that high expression leads to high activity is also evident in studies that use differential expression to identify microRNAs important in processes such as development or disease, which usually concentrate on microRNAs that are both highly and differentially expressed [23], [24]. This paradigm is supported by the observation that the over-expression of many microRNAs leads to dose-dependent decreases in the levels of target mRNAs [25], [26] and that the expression of microRNAs and their mRNA targets shows a weak negative correlation [27].

The relationship between microRNA activity and expression is not fully understood in any organism. Here we examine cellular levels of microRNAs and their repressive activities. Overall, we find that more highly expressed microRNAs repress their targets to a greater degree. However, microRNA* passenger strands are not less effective repressors than their dominant partner strands. We also identify microRNAs whose expression differ by 2 to 3 orders of magnitude and yet repress at the same level, and microRNAs with similar expression levels that exhibit a 30% difference in their repressive abilities. These findings demonstrate that microRNA cellular levels alone cannot be used as a reliable proxy for microRNA function, and that significant care is required when interpreting the likely functional consequences of a change in microRNA concentration.

## Materials and Methods

### Small RNA quantification

Total RNA was extracted from *D. melanogaster* S2-DRSC cells (Hillary Ashe, University of Manchester) using the Ambion miRvana kit according to the manufacturer's instructions. Small RNA library preparation, using the Illumina TruSeq Small RNA Kit, and Illumina sequencing were performed by GATC using a HiSeq 2000 machine. For ABI SOLiD sequencing, small RNAs were isolated using the Ambion FlashPAGE Fractionator according to the manufacturer's instructions and purified using the FlashPAGE reaction clean-up kit (Ambion). The ABI SOLiD library was constructed using the Small RNA Expression Kit (Life Technologies), according to the manufacturer's instructions, and sequenced using an ABI SOLiD 4 machine by the University of Manchester Genomics Core Facility. Illumina and SOLiD sequencing were each performed on two biological replicates. The sequencing datasets are deposited in the NCBI SRA database (accession number: SRS618054). MicroRNA microarrays were carried out by LC Sciences (miRBase version 17), also on two biological replicates. The array data are deposited in the GEO database (accession number: GSE58415).

### Ago-1 immunoprecipitation and RNA purification

Ago-1 immunoprecipitation was carried out in S2-DRSC cells. Cells were harvested and incubated with 200 µl lysis solution (Chromotek-GFP-Trap lysis buffer, 0.5 U/µl RNAse inhibitor (Roche) and 1× Protease inhibitor cocktail (Roche)) for 30 minutes on ice. Lysates were purified by centrifugation at maximum speed for 20 minutes at 4°C, and then transferred to Tosylactivated Dynabeads M-280 (Invitrogen) conjugated with 20 mg anti-Ago-1 antibody (ab5070). Samples were rotated at 4°C for 2 hours, followed by two washes, proteinase K digestion and RNA isolation as described in [28], with 1 U/µl RNAse inhibitor added to the buffers. RNA recovered after ethanol precipitation was used to prepare small RNA libraries with the Illumina TruSeq Small RNA Kit according to the manufacturers instructions. RNA qualities were analyzed with TapeStation and libraries were sequenced on the Illumina MiSeq platform generating 50 bp reads (University of Manchester Genomics Core Facility).

### Small RNA mapping

Illumina reads were trimmed using an in-house script and mapped to the *D. melanogaster* genome (assembly BDGP5). SOLiD sequencing reads were trimmed and mapped to the *D. melanogaster* genome using SeqTrimMap [29]. Reads mapping to predicted tRNAs (predicted using tRNAscanSE [30]) and annotated rRNAs (http://www.arb-silva.de/) were filtered from both datasets. The remaining short reads that map to the genome were then mapped to miRBase mature sequences (release 20) [31] using the Bowtie software 0.12.5 [32] with up to 2 mismatches in the first 17 nucleotides of the mature sequence and allowing any mismatch from the position 18 to the end of the mature sequence, in order to allow mapping of potential isoform reads. Table S1 details the total number of reads, total number of reads that map to the genome and the total number of reads that map to mature microRNAs. Normalized read counts for individual microRNAs are shown in Table S2. Sixteen pairs of mature/microRNA* sequences covering a range of expression levels were chosen for subsequent analysis. Mean read counts over the two biological replicates were normalized according to the total number of reads mapping to all mature microRNA sequences.

### Dual luciferase target assay

To generate the luciferase reporter plasmids, a single perfectly complementary microRNA target sequence was inserted into the XhoI site in the 3′ UTR of the *Renilla* luciferase gene in the psi-check-2 dual luciferase reporter construct (Promega). The firefly luciferase gene, also present in the plasmid, was used as a transfection control. 500 ng of plasmid was transiently transfected into 1×10^6^ S2-DRSC cells/ml in a 96 well plate using effectene (Qiagen). 48 hours later the cells were lysed using 40 ul 1× passive lysis buffer and subjected to the Dual-glo luciferase reporter system (Promega) and analysed using the MicroLumatPlus LB96V Microplate Luminometer. Three independent replicates were carried out on different days. The reported repression values are calculated as the ratio of *Renilla* luciferase to firefly luciferase activity, normalized to that of the empty reporter construct for each day of transfection. Raw values from the luminometer are shown in Table S3. A paired t-test was used to determine if suppression of the target reporter differed significantly from the empty reporter construct at a 0.05 significance level for each microRNA target site. Statistical analysis was carried out using the R software (R Development Core Team).

### MicroRNA target prediction

The complete set of Drosophila 3′ UTRs (release 5.52) was obtained from FlyBase. MicroRNA target sites were predicted using the 7 nt canonical seed method as described in [4]. Multiple target site predictions in different UTRs corresponding to the same gene were combined taking the maximal number of predicted target sites for each microRNA in the UTR set. Expression levels of the target mRNAs in S2-DRSC cells were extracted from [33]. The target site abundance for each microRNA was calculated as the sum of the targeted gene expression levels multiplied by the maximal number of target sites for a microRNA found in the UTR set for that gene. To determine whether insertion of the target sequences in the luciferase vector had inadvertently generated target sites for other microRNAs, we predicted canonical seed target sites for all *Drosophila* microRNAs (miRBase v20) in the inserted target sequences together with psi-check-2 sequences 6 nt upstream and downstream, using the canonical seed method. RNAhybrid [34] was used to predict the duplex stability of the mature microRNA sequences and their target sites.

## Results

### MicroRNA expression level is an unreliable indicator of microRNA repressive activity

We first sought to understand the relationship between endogenous microRNA expression and target repression. To this end, we estimated the relative abundance of microRNAs in *D. melanogaster* S2 cells using three distinct technologies: Illumina and ABI SOLiD deep sequencing, and microarray hybridization, each with two biological replicates. Although we find differences in the estimates of microRNA abundance, we focus on the trends that are consistent across all three technologies (see also Discussion). We present the analysis based on the Illumina dataset in the main text, and SOLiD and microarray results are shown in Supplementary Information. The normalized read counts for individual microRNAs from Illumina small RNA deep sequencing spanned 5–6 orders of magnitude. We chose a representative set of 16 pairs of *D. melanogaster* microRNAs (both presumed guide and passenger strands) that sampled the full range of expression and possessed low sequence similarity to minimize shared targeting with other microRNAs.

**Figure 1:**
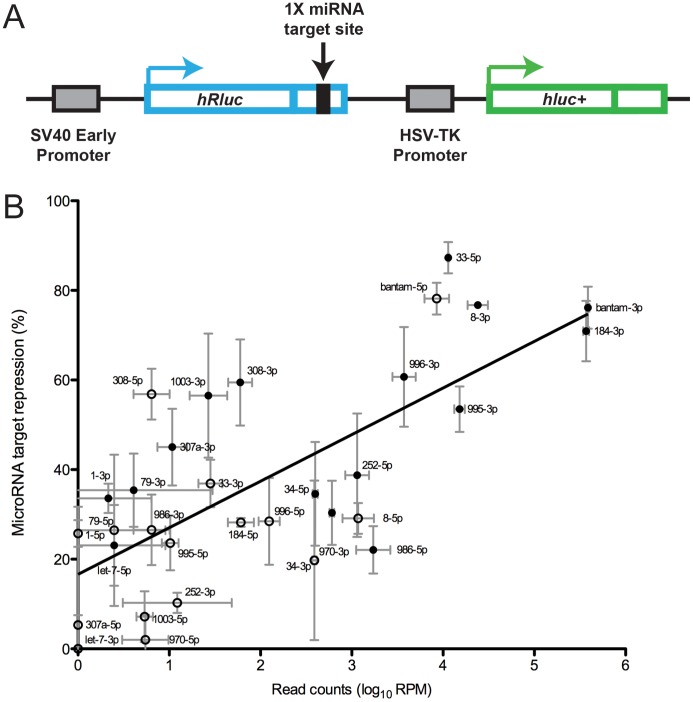
MicroRNA expression positively correlates with mRNA target repression. A) Schematic representing the vector used in repression assays containing *Renilla* luciferase gene, with a single microRNA targeting sequence incorporated into the 3′UTR, and the firefly luciferase gene used as the transfection control. B) Relationship of microRNA expression and target repression for 32 mature microRNAs. Repression of target mRNA was determined by dual luciferase reporter assay (see Methods). MicroRNA expression levels were estimated as normalized read counts (per million mapped to the genome – RPM) by Illumina small RNA deep sequencing on two independent samples of S2-DRSC cells – points represent average read counts. Filled points represent mature microRNA sequences and open points represent microRNA* sequences (defined as the less abundant arm when the ratio of arm abundance exceeds 4∶1). Error bars represent standard deviation. The line of best fit was estimated by linear regression.

To determine the repressive activity of our selected set of representative microRNAs on mRNA targets, we employed an *in vivo* assay to measure repression mediated by endogenous microRNAs in S2 cells. We specifically chose to investigate repression resulting from endogenous microRNAs so as to provide an accurate representation of *in vivo* activity. We generated 32 constructs using a single plasmid dual luciferase reporter system (psiCHECK-2 from Promega; Figure 1A). Modulation of mRNA translation by microRNAs has been demonstrated for both perfect and bulged target sites, with a moderate increased repression generally observed for perfect complementary sites [16], [35]. Each construct was engineered to contain a *Renilla* luciferase gene with a single, perfectly complementary microRNA target site incorporated into its 3′ UTR, and an untargeted firefly luciferase gene as a transfection control. These constructs were individually transfected into S2 cells and levels of *Renilla* and firefly luciferase were independently measured (Table S3). An active endogenous microRNA should result in a reduction of *Renilla* luciferase protein accumulation relative to the untargeted firefly luciferase. This allows us to correlate microRNA repressive activity with endogenous microRNA expression level.

As expected, normalized estimates of endogenous microRNA levels from Illumina deep sequencing were positively correlated with the level of repression of their targets, although the correlation is surprisingly modest (Pearson's correlation coefficient r = 0.43). The correlation between microRNA levels and target repression improves substantially (r = 0.71) when log_10_ transformed values of microRNA normalized read counts are used (Figure 1B; see Figures S1 and S2 for SOLiD and microarray data). We estimated the linear line of best fit between log_10_ normalized read counts and target repression. The gradient of this fitted line (10.4; 95% CI 6.6–14.2), suggests that, on average, a 10-fold increase in expression is needed to increase repression by approximately 10%. This relationship holds across microRNA expression levels that differ by 5–6 orders of magnitude. The level of repression of five low abundance mature microRNAs (let-7-3p, miR-307a-5p, miR-970-5p, miR-1003-5p, miR-34-3p) is not significantly different from the empty control vector (paired t-test; p<0.05). This is unsurprising and consistent with the expectation and observation that target repression depends on microRNA abundance. When we discard these sequences and repeat the analysis, the correlation between microRNA levels and target repression is essentially unchanged (r = 0.67; slope 8.7, 95% CI 6.7–10.7).

However, the level of repression exhibited by specific microRNAs varies greatly from the general trend. For example, there are multiple examples of microRNAs that have similar levels of expression but exhibit very different repression levels (Figure 1B). For example, miR-996-3p and miR-986-5p are expressed at similar levels, yet the former represses its target by over 60% while the latter induces less than 25% repression. Conversely, some microRNAs repress targets to similar degrees despite large difference in expression levels; for example miR-1-3p and miR-252-5p repress similarly yet have a more than 100 fold difference in expression levels. We also observe that the most abundant microRNAs (bantam-3p, bantam-5p, miR-184-3p, miR-33-5p, miR-8-3p) repress their targets by similar amounts, despite representing a more than 30-fold difference in expression. Even with these extremely high expression levels, we see no repression of luciferase translation greater than 80%; we therefore suggest that this represents a maximum achievable level of repression by endogenous levels of microRNAs.

Minor mature products (so-called microRNA* sequences; open points in Figure 1B) are defined by having lower abundance than their dominant mature microRNA partners. Unsurprisingly, the repression induced by microRNA* sequences is generally lower than that for the guide strands (filled points). However, the repression they mediate exhibits the same concentration vs. repression relationship observed for dominant products. In other words, microRNA* sequences in general repress their targets at approximately the level that is expected given their cellular concentration. We do however note two cases where the minor microRNA* product represses the target more effectively than the dominant arm (bantam and miR-986; compare open and filled points in Figure 1B), despite lower concentration in the cell.

In summary, we observe that microRNAs that similarly repress their targets can be expressed at levels that vary over several orders of magnitude. The data therefore clearly show that expression level is not a consistent determinant of repressive activity. Furthermore, almost all investigated microRNA* species are found to exert a repressive effect equivalent to the dominant mature sequences and in proportion to their cellular concentration. In some cases microRNA* strands are better repressors. These findings add significant weight to the growing view that mature sequences derived from both arms of the hairpin precursor have the potential to regulate targets in the cell; in other words, the two alternate mature sequences are not fundamentally different in functional consequence for protein production. To investigate these observations further, we sought to evaluate the influence of factors that might affect microRNA repressive efficiency.

### Additive repression of multiple microRNAs

We investigated the possibility that deviation from the expected repressive ability of a microRNA may be artifactual and due to additional microRNAs that target adventitious sites created at the boundaries of the engineered target site in the *Renilla* 3′UTR. To this end, we predicted canonical seed-match 7 nt target sequences (as described in [4]) for all *Drosophila* microRNAs in the region starting 6 nt upstream of the 5′ end of the inserted target sequence and ending at 6 nt downstream of its 3′ end in the luciferase vector, including the inserted sequence (see Table S4). We then summed the mean normalized read counts for all microRNAs that are predicted to repress the target site, and calculated the correlation with the observed target repression. Expression levels corrected to account for this additive effect correlate only marginally better with repression levels (r = 0.44 vs. r = 0.40). In the majority of cases however, the cloned target sites are not predicted to be targeted by other expressed microRNAs. We identify 9 cases where the repression of the target could be enhanced by the additional targeting from other expressed microRNAs (see Table S4). Just four of these cases (miR-307a-5p, miR-79-3p, miR-970-5p and miR-308-3p) are targeted by other microRNAs that are expressed at similar or higher levels than the microRNA for which the assay was designed. Figure 1B shows that miR-79-3p and miR-308-3p are repressed more than expected from the expression level of the specific targeting microRNA alone. We now suggest the increased repression is likely to be due to repression by other microRNAs targeting adventitious sites generated in cloning the target site into the vector.

### Target abundance

It has been predicted that a typical microRNA has approximately 100 targets [36] but this number can vary hugely for different microRNAs. It is therefore conceivable that microRNA activity may depend on the number of target sites for a given microRNA in a cell. For example, a large amount of targeted mRNA could sequester microRNA/RISC, and other targets would therefore be less effectively repressed. Therefore, the levels of expression of targeted mRNAs could have an impact on the availability and consequently the level of microRNA repressive activity. To investigate this relationship, we calculated a measure of the number of target sites for a given microRNA in the cell, and compared with microRNA repressive ability. We predicted canonical seed microRNA targets in 3′UTRs of all FlyBase transcripts and obtained expression levels of the target mRNAs from tiling array data [33]. The number of target sites was then approximated as the sum of the mRNA target abundances multiplied by number of predicted target sites within each mRNA. We find no significant correlation between number of target sites and the ratio between microRNA repression levels and expression levels (r = −0.09) (Figure S3). We therefore find that target abundance does not significantly affect microRNA repression efficiency in this assay.

### MicroRNA/target duplex stability

**Figure 2:**
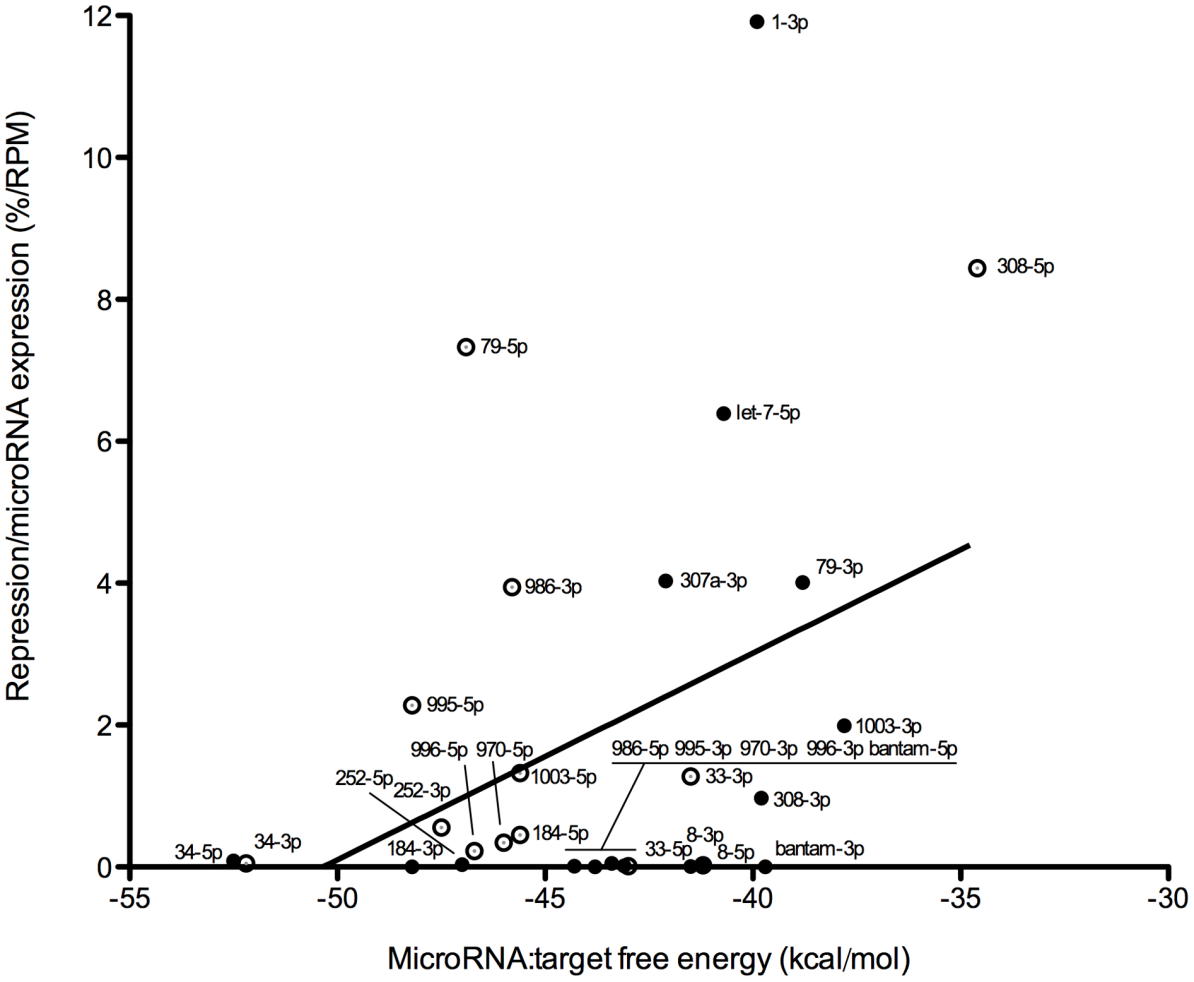
Relationship between predicted free energy of the microRNA:target duplex and expression-corrected microRNA repression. The free energy of 32 chosen microRNA duplexes was predicted by RNAhybrid. Expression-corrected microRNA repression was calculated as the ratio of the repression level for a given microRNA and its averaged normalized log_10_ read counts.

In order to determine whether the stability of the microRNA/target duplex influences repression level, we predicted the free energy of each duplex using RNAhybrid. A number of microRNA/target duplexes display low stability and high repression per expressed copy, including let-7-5p, miR-79-3p, miR-1-3p, miR-308-5p and miR-308-3p. The microRNAs that exhibit the greatest duplex stability show very low repression. Indeed, there is a weak correlation (r = 0.39) between free energy and expression-corrected microRNA repression, calculated as the ratio of microRNA repression and log transformed microRNA abundance (Figure 2); i.e. duplex stability and expression-corrected repression are negatively correlated. The GC content of a microRNA highly influences duplex stability, therefore a correlation exists between microRNA GC content and repression relative to expression (r = −0.35). The data therefore show that microRNA/target duplex stability influences but does not explain microRNA repressive ability.

### Association of microRNAs with Argonaute proteins in the RISC

**Figure 3:**
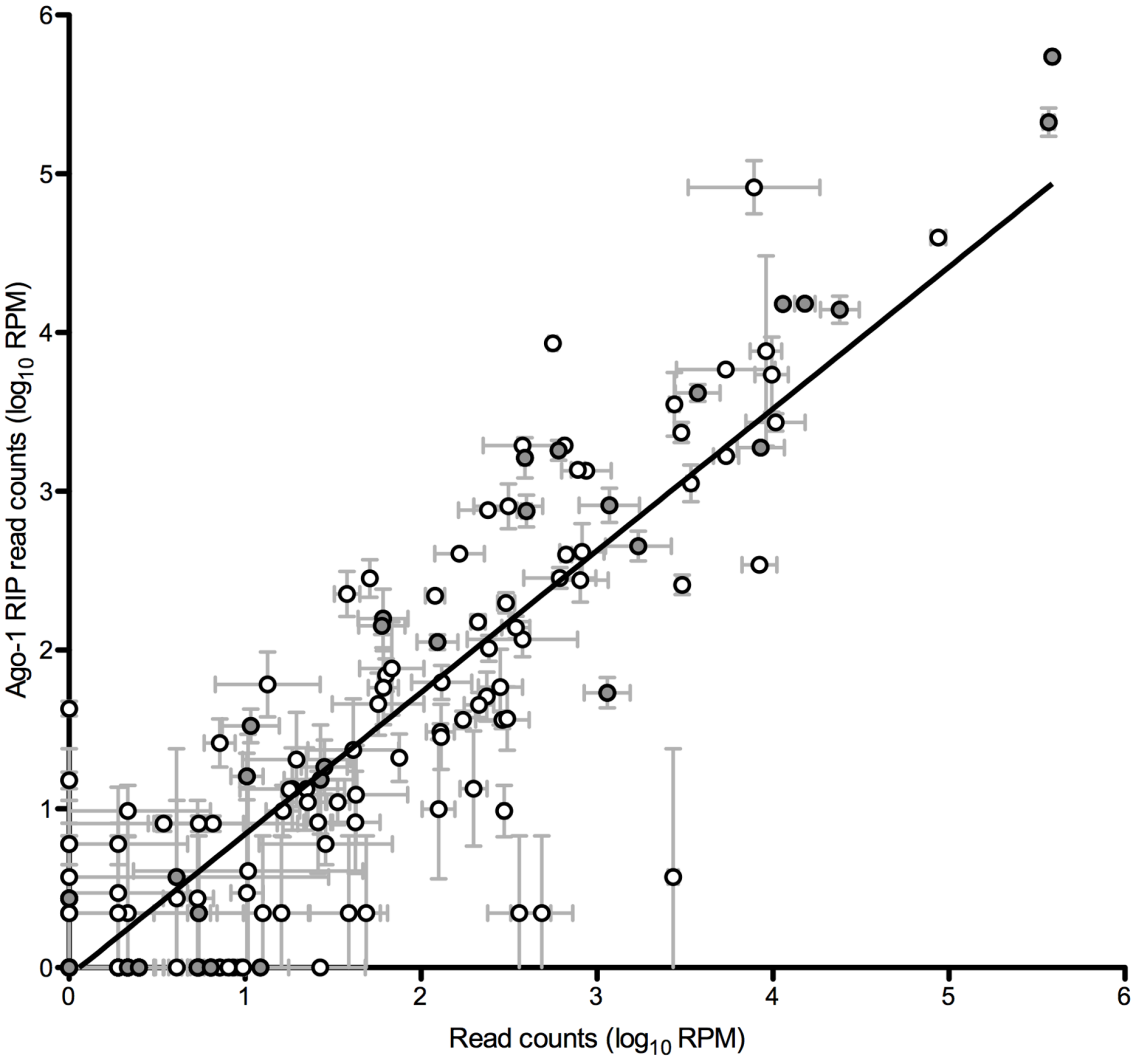
Correlation between microRNA levels in the cell and in Ago-1 immunoprecipitation experiments. MicroRNA expression levels were estimated by Illumina deep sequencing on two independent samples of size-fractionated RNA from S2-DRSC cells for both cellular and Ago-1 pull-down. Filled circles represent the microRNAs chosen for further analysis. The x-axis shows log_10_ normalized cellular read counts expressed as average reads per million, for two samples. The y-axis shows log_10_ normalized Ago-1 pull-down read counts expressed in reads per million, for two independent samples. Line of best fit was produced by linear regression.

**Figure 4:**
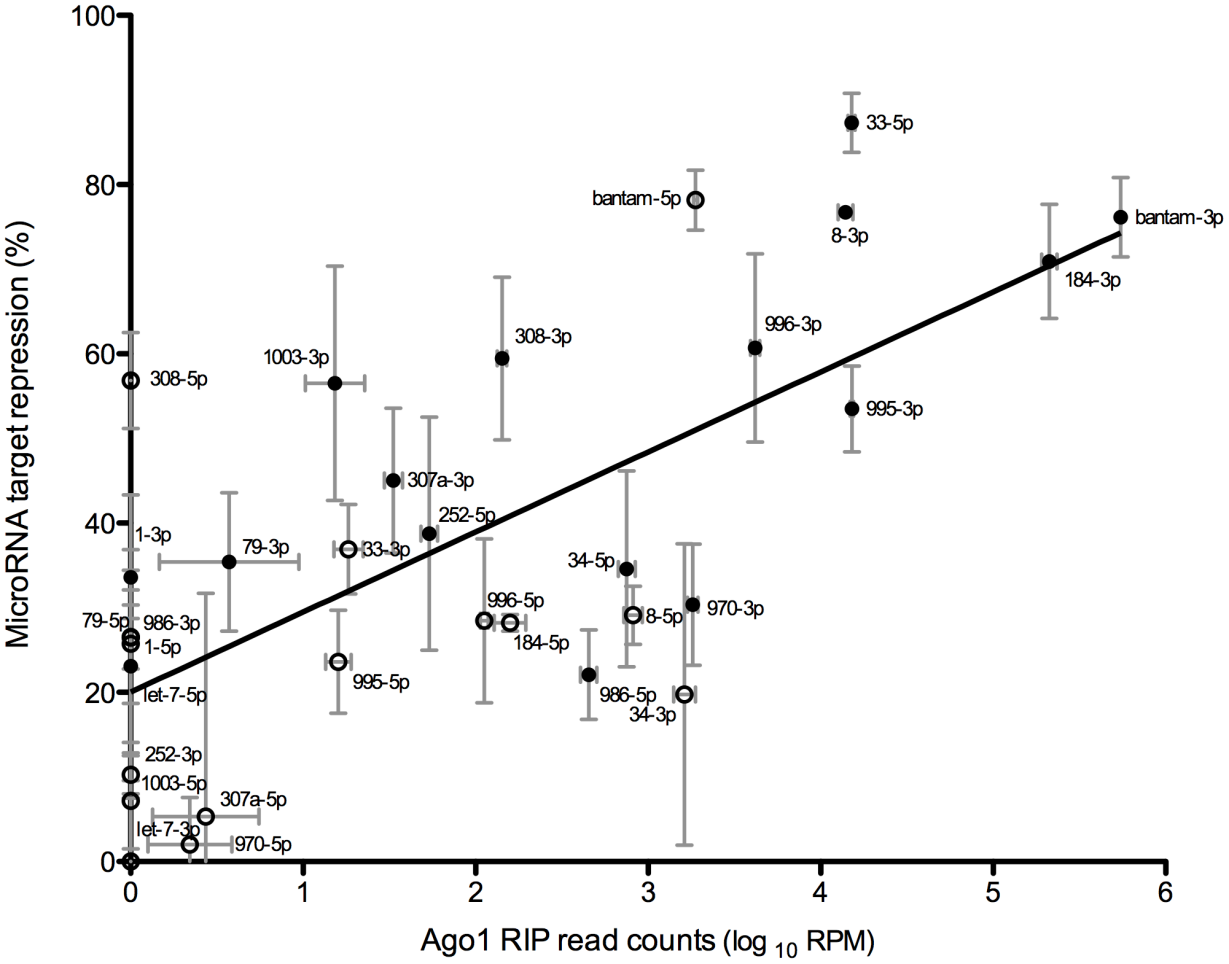
Ago-1 pull-down microRNA levels correlate positively with microRNA repression levels. Repression of target mRNA was determined by dual luciferase reporter assay containing microRNA target sites, for 32 chosen microRNAs. Ago-1 pull-down microRNA levels were obtained through Illumina deep sequencing on two independent samples of S2-DRSC cells. The read counts are normalized (reads per million – RPM) and averaged across 2 sequenced samples. Filled circles represent mature microRNA sequences and open circles represent microRNA* sequences (defined as the less abundant arm when the ratio of arm abundance exceeds 4∶1). Error bars represent the standard deviation. The line of best fit is calculated by linear regression.

MicroRNA function requires physical association with the Ago-1 protein in the RISC [11], [37]. Differential RISC occupancy may therefore be the crucial step modulating target repression. To determine whether Ago-1 associated microRNAs are an improved determinant of microRNA activity, we performed two independent Ago-1 RNA immunoprecipitation experiments and estimated microRNA association using Illumina sequencing. Figure 3 shows the relationship between normalized microRNA read counts from the Ago-1-RIP experiments and the microRNA cellular expression levels. Globally, cellular levels of microRNAs correlate extremely well with Ago-1 associated microRNA levels (r = 0.98). However, there are numerous examples of microRNAs that are present in RISC more than expected from their expression levels (miR-14-3p, miR-317-3p, 275-3p) or less than expected (miR-13a-3p, miR-317-5p, miR-190-3p). MicroRNA association with Ago-1 therefore differs amongst microRNAs, suggesting that occupancy in the RISC is differentially regulated, and could account for some of the discrepancies observed in the repressive ability of individual microRNAs. However, we find that the correlation of Ago-1 microRNA association with microRNA repression is very similar (r = 0.69; Figure 4) to cellular microRNA levels versus microRNA repression (r = 0.71; Figure 1B); in other words, microRNA repression is not better explained by the amount of microRNA associated with the RISC. Furthermore, it has been shown that Ago association is influenced by many different factors, including the 5′ nucleotide of the microRNA, microRNA duplex stability, and stability of the microRNA/target duplex [10]–[14], [38]–[40]. We find no significant correlation between any of these factors and microRNA occupancy in the RISC (as measured by the ratio of Ago-1 and cell read counts; data not shown).

## Discussion

### Using microRNA expression level as a proxy for activity

MicroRNAs have been predicted to be responsible for regulating a large proportion of protein coding genes involved in a wide variety of cellular processes [41], [42]. Although little is known about how the expression of an individual microRNA is related to its activity in vivo, it is a commonly-held view that microRNA expression levels can be used as a proxy of their activity [21], [26], [33]. The correct interpretation of all experimental work that uses the microRNA machinery to manipulate gene function requires an understanding of this relationship.

Overall, we find the relationship between microRNA expression and repression is reasonably well described by a log-linear fit, with a 10-fold increase in expression required for approximately 10% increase in repression. This relationship holds across the entire range of endogenous microRNA expression levels in *Drosophila* S2 cells. Therefore, to achieve a fixed linear increase in repression of a microRNA target will require an exponential increase in microRNA concentration. This is consistent with the repeated observation that increases in microRNA concentration often have relatively mild effects on the repression of target mRNAs [15], [16], [35], [43]. Our observations suggest that a 2-fold change in microRNA expression (a common threshold used to identify significant changes in differential expression analysis) would lead to changes in microRNA target levels of only a few percent.

Our analysis depends on the accurate estimation of microRNA abundance and target repression. It has been previously reported that different expression technologies have different biases [44]. We therefore estimated microRNA expression using three different platforms – Illumina and ABI SOLiD sequencing, and a microarray hybridization – on the same samples of S2 cell small RNAs (see Table S1 and S2). Illumina and SOLiD sequencing involve completely different chemistries – the former is sequencing by synthesis and the latter by ligation. We might therefore expect any sequence-specific biases introduced by the methodologies to be different. As expected, we find the estimates of abundance of some microRNAs differ between the three platforms. However, the relationship between abundance and repression across a large number of microRNAs holds regardless of the technology used to estimate microRNA levels (see Figures S1 and S2). Indeed, we find that the estimates of the repressive effects of a 10-fold increase in microRNA abundance from ABI SOLiD and microarray datasets fall within the 95% confidence interval calculated from the Illumina dataset. The reproducibility of expression estimates between replicate datasets also differs between technologies (see Figure S4), with the array data significantly more noisy than the two sequencing technologies. The Illumina data is the most reproducible between replicates, and so we chose to focus on that technology for the Ago ChIP-seq experiment and further analysis.

A number of factors are known to influence microRNA activity, including number of target sites per mRNA and the degree of complementarity [45], [46]. For example, repression is affected by varying degrees of complementarity between the microRNA and its target, but to different extents with different microRNAs. Multiple copies of microRNA target sites in the UTRs of reporter constructs have been shown to enable greater levels of repression, and cooperativity has been demonstrated for closely positioned microRNA target sites [47]. We have attempted to remove the complexity and variability between different microRNAs and target contexts by examining a constrained case where targets have a single perfectly complementary site in a homogeneous cell population. The rate at which the microRNA binds to and dissociates from the target is likely to be slower with these perfectly complementary sites than for seed-match targets [48]. However, it has been shown that reporter assays with fully complementary targets and mismatched targets exhibit similar patterns of repression [15], [16].

Previous studies have investigated aspects of the relationship between microRNA abundance and microRNA activity in a variety of systems [15], [16], [35], [43], [49]. For example, Yang et al. over-expressed microRNAs in vertebrate cells to investigate the relationship between microRNA expression and repressive ability [16]. We have chosen instead to study the repressive effects of endogenous levels of microRNAs to eliminate possible issues with differences in microRNA delivery and toxicity at high levels. Some previous studies have also concluded that a threshold level of a microRNA is required for the repression of a target, and that only the most abundant microRNAs effectively repress their targets [15], [49]. Our data do not directly contradict this view, but we demonstrate clear and measurable repressive effects of microRNAs expressed at 3 to 4 orders of magnitude below the maximum observed endogenous microRNA levels in S2 cells. Indeed, on average, the log-linear relationship between repression and microRNA abundance appears to hold across microRNA expression levels covering 5–6 orders of magnitude.

The log-linear relationship between microRNA expression and repression suggests that there are factors buffering the function of expressed microRNAs, acting on the saturation of a step or component of the overall reaction. There are two apparent candidates for that limiting step: the loading of the microRNA into the RISC complex, and the binding or association of the loaded RISC to the mRNA target. We show that microRNA expression and the levels of microRNAs associated with Ago-1 show a linear relationship across the entire range of microRNA concentrations, suggesting that in general the level of association of a microRNA with the RISC is determined by its cellular abundance; i.e. the association of a microRNA with the RISC is not limiting. We therefore speculate that the dynamics we observe suggest a bottleneck at the point of association and action of RISC with a target mRNA, either due to saturation of target sites on mRNAs, the rate of target site identification, or the proportion of binding events that result in repression. It has been shown that *Drosophila* Ago-2 action is not limited by target site identification and initial RISC binding [48]. If target sites of highly expressed microRNAs are nearly completely occupied, a moderate increase in repression would require a massive increase in the number of microRNA molecules in the cell. This may help explain how microRNAs fine tune protein production as well as buffering protein levels, yet still allowing transcriptional regulation to dynamically modulate mRNA levels [43], [45].

### Both microRNA and microRNA* sequences are effective repressors

For the majority of microRNAs, one arm of the microRNA duplex accumulates to a much greater level than the other [21], [22]. This is exemplified by the bantam microRNA, which is highly expressed in S2 cells and is involved in a number of important cellular processes such as cell proliferation, apoptosis, development and the circadian clock [50], [51]. The two possible mature microRNAs are found at approximately 50-fold difference in abundance. However, both sequences are effective repressors, with the less abundant -5p sequence actually being the more active repressor. Indeed we observe two microRNAs in which the less abundant strand is a more active repressor of the target sequence, and on average, the less abundant microRNA* sequences tested repress their targets at levels consistent with their expression and similar to their partner dominant strand. The identification of functional capabilities of the non-dominant mature microRNA impacts on work on the identification of targets; for example, some of the observed function of bantam may be attributable to the non-dominant -5p sequence.

### Multiple factors influence microRNA activity

After controlling for the generation of adventitious target sites in the luciferase vector for other microRNAs expressed in S2 cells, only six microRNAs are found to repress their targets at levels greater than expected (bantam-5p, miR-33-5p, miR-8-3p, miR-307a-3p, miR-308-5p and miR-8-3p). However, our analysis identifies a number of microRNAs that repress their targets less than would be expected from their expression levels. We therefore suggest the existence of unidentified limiting factors or processes that down-regulate some microRNA activities, and that there is no evidence for increased microRNA activity above the observed abundance/repression relationship. Recent reports have suggested that target availability may influence microRNA activity in relation to expression, in that a microRNA that represses more abundant targets would be diluted from the pool of available microRNAs [52]. However, other work contradicts this observation as it has been shown that increasing concentrations of target do not easily saturate microRNA repressive ability [15]. Indeed, a recent study of the kinetics of target repression for the let-7 microRNA suggests that only microRNA/target pairs with a very narrow range of expression levels are susceptible to perturbation of their activity by decoy RNAs [48]. Our data for 32 mature sequences across the complete range of expression levels is in agreement with the latter observation, as we do not observe any correlation between microRNA repressive ability and number and expression levels of predicted targets. These two observations together suggest that the dynamics of target/RISC association is limiting across all concentrations.

Using our dataset of small RNA sequences from an Ago-1 IP experiment, we show that the efficiency of incorporation of microRNAs into the RISC is relatively consistent and mostly dependent upon microRNA expression levels. Repression correlates similarly with abundance in the Ago-1 dataset and microRNA expression level, and so the rate of Ago-1 association is unable to explain variations in microRNA repression. Argonaute association has been reported to be dependent upon a number of factors including microRNA nucleotide composition, and thermodynamic stability of microRNA duplexes and microRNA:target complexes [10]–[14], [38]–[40]. Though these factors are undoubtedly relevant, our data do not support a significant correlation of any of these factors with microRNA repressive ability. We do, however, see a weak negative correlation between microRNA/target duplex stability and repressive ability. A single microRNA/RISC complex has been shown to be able to catalyse the cleavage of a number of target molecules [53]. Lower stability of the microRNA/target duplex may therefore favour transient interaction with target mRNAs, thus enabling higher processivity of the microRNA and efficient down-regulation of multiple targets.

Our microRNA expression level measurements assess steady-state levels, which are obviously affected by the balance of production and degradation. Indeed, the half-life of a microRNA will likely have a significant effect on its activity. It has been reported that the stabilities of individual microRNAs are differentially regulated [54]. Translin, a DNA/RNA binding protein, has been shown to increase the stability of miR-122, but not other microRNAs, in mice testis. A subset of microRNAs may therefore be bound by RNA binding proteins to alter their stability and consequent repressive ability. Other factors that may influence microRNA stability and activity include methylation, 3′ nucleotide additions such as uridylation, and the expression of 3′ to 5′ exonucleases [55]–[59].

Our analyses show that microRNA abundance is positively correlated with repression, but that the relationship is not a straightforward one. We also show that microRNAs expressed at levels differing by 2 to 3 orders of magnitude may elicit similar repressive effects. The ability to predict the magnitude of repression solely from the abundance of a mature microRNA is therefore limited. In particular, we find that microRNA* products often repress targets just as well as, and sometimes better than, the dominant guide strand mature microRNAs. We demonstrate that microRNA/target duplex stability regulates microRNA activity, but it is not sufficient to explain discrepancies between cellular abundance and target repression. The differential association of microRNAs with Ago-1 does not better explain repressive ability, and so we suggest that target repression is subject to unknown factors that down-regulate microRNA activity. On average, the relationship between expression and repression suggests that a 10 fold increase in microRNA abundance is required to produce an approximately 10% increase in repression. This is consistent with a model where microRNA activity is subject to extensive buffering and the rate-limiting step in microRNA activity is at the level of association of the target mRNA with microRNA-loaded RISC complex.

## Supporting Information

Figure S1 **MicroRNA expression (SOLiD read counts) positively correlates with mRNA target repression.** Relationship of microRNA expression and target repression for 32 mature microRNAs. Repression of target mRNA was determined by dual luciferase reporter assay (see Methods). MicroRNA expression levels were estimated as normalized read counts (per million mapped to the genome – RPM) SOLiD small RNA deep sequencing on two independent samples of S2-DRSC cells – points represent average read counts. Filled points represent mature microRNA sequences and open points represent microRNA* sequences (defined as the less abundant arm when the ratio of arm abundance exceeds 4∶1). Error bars represent standard deviation. The line of best fit was estimated by linear regression (r = 0.68).
(DOCX)

Figure S2 **MicroRNA expression (microarray fluorescence) positively correlates with mRNA target repression.** Relationship of microRNA expression and target repression for 32 mature microRNAs. Repression of target mRNA was determined by dual luciferase reporter assay (see Methods). MicroRNA expression levels were estimated as normalized Cy3 fluorescence for two independent hybridizations of RNA from S2-DRSC cells. Filled points represent mature microRNA sequences and open points represent microRNA* sequences (defined as the less abundant arm when the ratio of arm abundance exceeds 4∶1). Error bars represent standard deviation. The line of best fit was estimated by linear regression (r = 0.36).
(DOCX)

Figure S3 **Relationship between predicted microRNA target site levels and expression-corrected microRNA repression.** Predicted microRNA target site levels are calculated as the sum of the maximum number of 7 nucleotide seed regions in the 3′UTRs of all targets of a microRNA multiplied by the mRNA expression. Expression-corrected repression is determined from the ratio of repression to Illumina small RNAseq read counts. Closed circles represent the 32 microRNAs examined in this study.
(DOCX)

Figure S4 **Replicate correlation for microRNA abundance estimates.** MicroRNA abundance was estimated using Illumina deep sequencing (A), ABI SOLiD deep sequencing (B), and microarray hybridization (C). The X and Y axis represent the abundance estimate for two biological replicates. Correlation coefficients are present in the upper right of each panel.
(DOCX)

Table S1 **Summary statistics for deep sequencing and mapping of small RNAs.** Data are presented for two biological replicates for Illumina, ABI SOLiD, and Illumina sequencing of Ago-1-RIP experiments. Read counts mapping to genome and read counts mapping to microRNAs are shown as raw numbers and percentage of total read counts. The low percentage of small RNAseq reads mapping to microRNAs is due to the high abundance of the Drosophila-specific 30 nt 2S ribosomal RNA.
(XLSX)

Table S2 **Normalized Illumina and SOLiD read counts mapping to microRNAs.** Read counts were normalized according to the total number of reads mapping to all mature microRNA sequences (expressed as reads per million). Illumina and SOLiD sequencing was carried out on the same two total RNA samples in order to estimate variability between the two sequencing platforms.
(XLSX)

Table S3 **Raw luciferase read counts.** Firefly and *Renilla* luciferase genes are both contained in the same plasmid. Firefly luciferase is used as a transfection control. *Renilla* luciferase contains a single copy of the chosen microRNA target site in the 3′UTR. Table displays raw luciferase counts, the ratio between Renilla and Firefly luciferase, calculated expressiona dn calculated expression for each of our chosen microRNAs. See methods section for experimental details.
(XLSX)

Table S4 **Design and properties of microRNA target constructs.** The columns show the names of microRNAs examined in this study, assignment of */non-star, mature microRNA sequence, sequence of microRNA target site with flanking 6 nt downstream and 6 nt upstream vector sequence (target sequence in bold), list of additional microRNAs targeting at the cloning junction together with their cellular expression levels (average RPM between two sequenced samples) and the predicted free energy of the microRNA-target duplex. Seed sequences of additional microRNAs potentially binding the target site are shown in column 4 (underlined).
(XLSX)
