# Supplementary figures and images for: Target Repression Induced by Endogenous microRNAs: Large Differences, Small Effects
Source: PLoS One. 2014 Aug 20;9(8):e104286. doi: 10.1371/journal.pone.0104286 (PMC4139194; doi:10.1371/journal.pone.0104286)

**Figure S4**

**
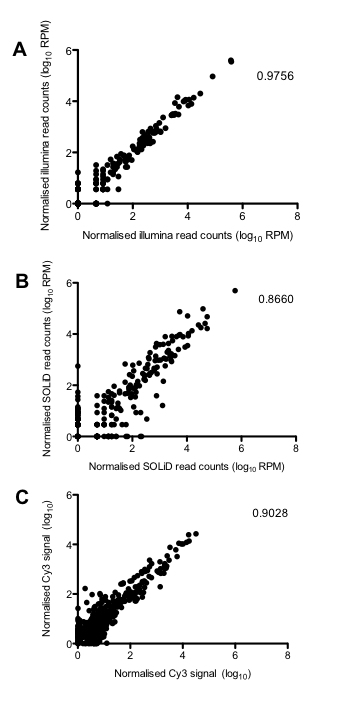
**

Supplement: Figure S4 — Replicate correlation for microRNA abundance estimates. MicroRNA abundance was estimated using Illumina deep sequencing (A), ABI SOLiD deep sequencing (B), and microarray hybridization (C). The X and Y axis represent the abundance estimate for two biological replicates. Correlation coefficients are present in the upper right of each panel. (DOCX) [file pone.0104286.s004.docx]
